# Biomechanical Model-Based Development of an Active Occupational Upper-Limb Exoskeleton to Support Healthcare Workers in the Surgery Waiting Room

**DOI:** 10.3390/ijerph17145140

**Published:** 2020-07-16

**Authors:** Mark Tröster, David Wagner, Felix Müller-Graf, Christophe Maufroy, Urs Schneider, Thomas Bauernhansl

**Affiliations:** 1 Fraunhofer Institute for Manufacturing Engineering and Automation IPA, Nobelstraße 12, 70569 Stuttgart, Germany; david.wagner@ipa.fraunhofer.de (D.W.); felix.mueller-graf@ipa.fraunhofer.de (F.M.-G.); christophe.maufroy@ipa.fraunhofer.de (C.M.); urs.schneider@ipa.fraunhofer.de (U.S.); thomas.bauernhansl@ipa.fraunhofer.de (T.B.); 2 Institute of Industrial Manufacturing and Management IFF, University of Stuttgart, Allmandring 35, 70569 Stuttgart, Germany

**Keywords:** healthcare, ergonomics, manual work, exoskeleton, musculoskeletal modeling

## Abstract

Occupational ergonomics in healthcare is an increasing challenge we have to handle in the near future. Physical assistive systems, so-called exoskeletons, are promising solutions to prevent work-related musculoskeletal disorders (WMSDs). Manual handling like pushing, pulling, holding and lifting during healthcare activities require practical and biomechanical effective assistive devices. In this article, a musculoskeletal-model-based development of an assistive exoskeleton is described for manual patient transfer in the surgery waiting room. For that purpose, kinematic data collected with an experimental set-up reproducing real patient transfer conditions are first used to define the kinetic boundary conditions for the model-based development approach. Model-based analysis reveals significant relief potential in the lower back and shoulder area of the musculoskeletal apparatus. This is corroborated by subjective feedback collected during measurements with real surgery assistants. A shoulder–arm exoskeleton design is then proposed, optimized and evaluated within the same simulation framework. The presented results illustrate the potential for the proposed design to reduce significantly joint compressions and muscle activities in the shoulder complex in the considered patient transfer scenarios.

## 1. Introduction

Prevention of WMSDs, the main cause of sickness-related work absenteeism in many developed countries (23.4% in Germany [1] and 52% in Europe [2]), is a pressing challenge regarding occupational ergonomics, especially in the context of the ongoing demographic changes leading to the aging of the workforce. This is especially important in the healthcare sector, which is already facing a shortage of qualified personal and where nursing staff must often carry out physically demanding activities, such as patient handling in strenuous postures. A recent systematic review among nurses reported for instance a high prevalence (71.85%) of WMSDs [3], especially for the lower back, shoulder and neck area. High WMSD incidence was also found among radiologists [4], while [5] also reported high prevalence of WMSDs among dentists in Germany (about 86.7% of them stated to suffer from pain in the neck and shoulder region).

Ergonomic interventions, including the optimization of work posture and conditions, obligatory muscle and movement training, regular work breaks and load-specific work management are possible means to decrease the burden on the musculoskeletal system, enabling a longer, healthier and happier working life for the affected employees. Leading specialists from the World Health Organization (WHO) formulated for example in [6] a list of practical and preventive instructions. If the pursued ergonomic interventions alone are not sufficient according to the recommended ergonomic evaluation methodologies, further approaches involving mechanical assistive devices must however be considered. Beside traditional devices, such as patient lifters, slide sheets, transfer boards, slings, belts or rail systems and a new type of wearable assistive devices, called exoskeletons, have emerged recently as promising systems to alleviate the physical strain of their wearer, while being more flexible, dynamic and less time-consuming in usage. Through external mechanical structures strapped to the user body, exoskeletons can apply assistive torques to support the wearer’s musculoskeletal apparatus. The so-called *occupational exoskeletons* [7] are more particularly intended for worker support and aim at decreasing muscle activity and joint compression forces in highly loaded body regions, for instance when handling heavy loads or working in strenuous body postures.

**Figure 1:**
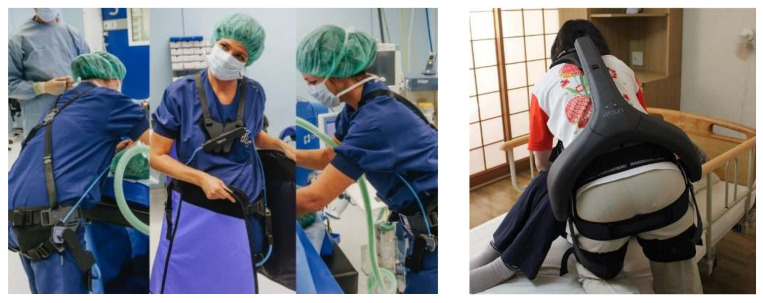
Exemplary devices are passive (see left photo, *Laevo* [16]) and active (see right photo, *Atoun Model Y* [14]) exoskeletons applied so far for healthcare purposes.

The last decade has seen an increasing number of commercial and research initiatives addressing the development of *occupational* exoskeletons [8,9]. *Occupational exoskeletons* can be either passive or active; passive exoskeletons only use unpowered mechanisms such as a spring to generate the supporting forces while active ones involve powered force/torque generating elements such as motors [7]. Most of the occupational exoskeletons are intended for usage in industrial applications, such as in the automotive industry for overhead assembly (exemplary devices are *Skel’Ex*, *Paexo Shoulder*, *Levitate*, *Mate* and *ShoulderX* [9]) or in the logistics for parcel handling (exemplary devices are *SoftExo* [10], *Paexo Back* [11] and *Cray X* [12]). Only a limited number of them were considered so far for caretaker support in healthcare applications. These include mostly hip support exoskeletons such as the passive exoskeleton *Laevo* [13] for static holding applications in surgery (see Figure 1) and the *Atoun Model Y* [14] for manual patient handling and other daily living healthcare applications. Regarding upper limb support, [15] evaluated for instance a passive upper-limb exoskeleton in a static laparoscopy operation procedure in the lab and test operation environment. Pain and fatigue in the shoulder area were considerably reduced without significantly interfering with operative skills or manual dexterity.

**Figure 2:**
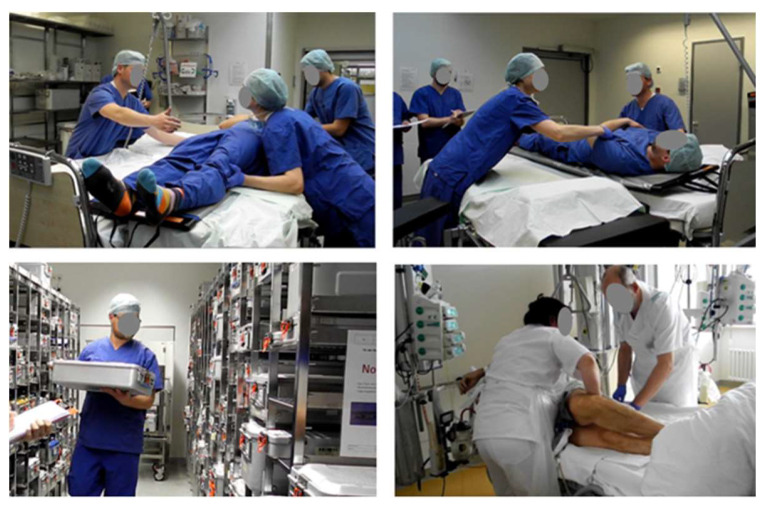
Typical manual handling scenarios identified and investigated in a preliminary workshop with hospital personal. Upper left: manual patient transfer in the post-op waiting room, upper right: manual patient transfer in the pre-op waiting room, lower left: manual handling in the logistics operation area, lower right: manual patient transfer in the intensive care unit (Diakonie-Klinikum Stuttgart, photos taken on 28 March 2019).

In this context, the *ExoPflege* project [17] is aiming at developing an *occupational exoskeleton* designed to support the nursing staff working in the surgical department, where an increased risk of WMSDs has been reported [3]. Among the four manual handling work scenarios identified and investigated in preliminary workshops with hospital personal (Figure 2), manual patient transfer in the post-op and pre-op waiting rooms were identified as the most critical ones from the ergonomics perspective. In these scenarios, caretakers carry out mainly horizontal pushing and pulling movements, for which, to the author’s best knowledge, no suitable exoskeleton has been developed so far. The present paper reports the first steps towards the development of such a wearable device. For that purpose, a biomechanical model-based framework previously set up by the authors and used for the analysis of human–exoskeleton coupled systems in the context of industrial *occupational exoskeletons* [18] is leveraged to design, optimize and evaluate a novel active upper-limb exoskeleton concept.

First in the article, a laboratory analysis is described for the selected caretaker’s manual handling scenarios, further a model-based biomechanical analysis of the scenarios is outlined. In parallel, the subjective feedback of the investigated caretakers is analyzed to indemnify the biomechanical objective results. In the next section, the optimization of an active shoulder–arm mechanism is described and further evaluated based on the measured manual pushing and pulling. Discussion, conclusions and future work are finally outlined to learn from the applied approach for future exoskeleton developments.

## 2. Methods

### 2.1. Laboratory Analysis

During the workshop, subjective feedback from experienced personal was collected and discussed to clarify the boundary conditions of the application scenarios. Amongst others, the researchers collected geometries of each application environment, times of processing and repetition and handling movement strategies. For the scenarios selected as ergonomic critical after the evaluation of the workshop results, mock-ups as application replications were set up in the laboratory.

To approximate the biomechanical strain based on a musculoskeletal model, the surgery assistants’ body motions, ground reaction forces and bed reaction forces were measured. Kinematics were collected by attaching 41 markers on the segments of the body of the subjects and using 12 optical motion capture cameras (Qualisys) with a sampling frequency of 100 Hz to capture full-body movement. The cameras were adjusted on the relevant task area in order to capture the motion precisely and to reduce the risk of occluded markers. Polynomial spline interpolation was used during post-processing in order to fill the gaps in the marker trajectories, which were a result of marker misdetections during measurement. Furthermore, ground reaction forces and reaction forces from leaning against the hospital bed were collected using 6-axis force plates (AMTI) and 6-axis force-torque sensors (Sensix).

**Figure 3:**
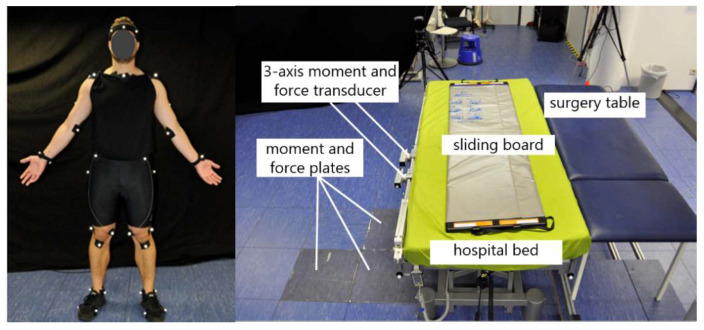
Full-body labeled test person (left side) and application mock-up (right side) in the biomechanical laboratory with integrated force and moment sensors to detect external loads.

Data were collected with three surgery assistants (2 male/1 female, age 32.5 +/− 2.5) within five different trials. During each run, the kinematics of the subject performing the patient transfer was recorded using a full-body marker set (see Figure 3). The other subjects assisted the procedure to simulate as realistically as possible the manual handling scenarios (their motions were however not recorded). In all measurements, a 35-year old male (93 kg) was playing the role of the patient. Feedback about the local body strain experienced by the subjects during the transfer procedure was also collected within a questionnaire based on the Borg-Scale, for later comparison with the results of the model-based biomechanical analysis.

### 2.2. Model-Based Biomechanical Analysis

Using the musculoskeletal modeling tool AnyBody Modeling System (AMS) in version 7.2, the biomechanical analysis was executed. AMS is capable of analyzing rigid multi-body systems like the musculoskeletal system of the human or other creatures. In addition, AMS is capable of including external objects, loads and in vivo measured motion data to compute inner body torques and forces through an inverse dynamic approach. Having motions and external forces as measured input, AMS solves the dynamic equilibrium equations [19].

**Figure 4:**
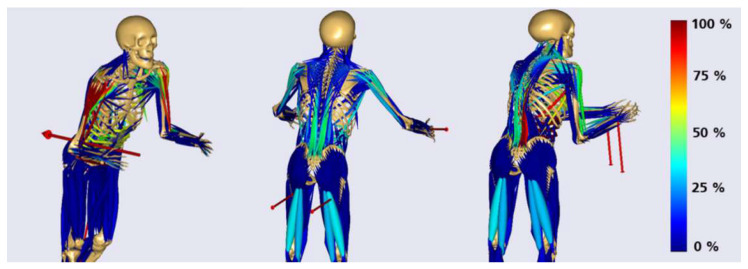
Musculoskeletal models in high loaded static postures with visualization of muscle activation (left: pushing, middle: pulling and right: lifting), the models are shown with external body forces as red arrows in the representative posture for the three application scenarios.

Based on motion and force data of the lab measurements, excessively loaded postures were identified. By solving a static equilibrium of measured ground and bed reaction forces, external hand forces were approximated as boundary conditions. However, it was assumed that both hand contact forces had the same magnitude in each posture. For the overall analysis approach the assumption of symmetric hand loading was assumed to simplify application-specific biomechanical effects, detailed effects of asymmetric hand loading can be further investigated in combined exoskeleton–human models. The load postures were selected by considering the highest measured hand forces within each application´s and subject´s trial. Based on these assumptions three representative static load models for each application scenario were built (see Figure 4).

**Figure 5:**
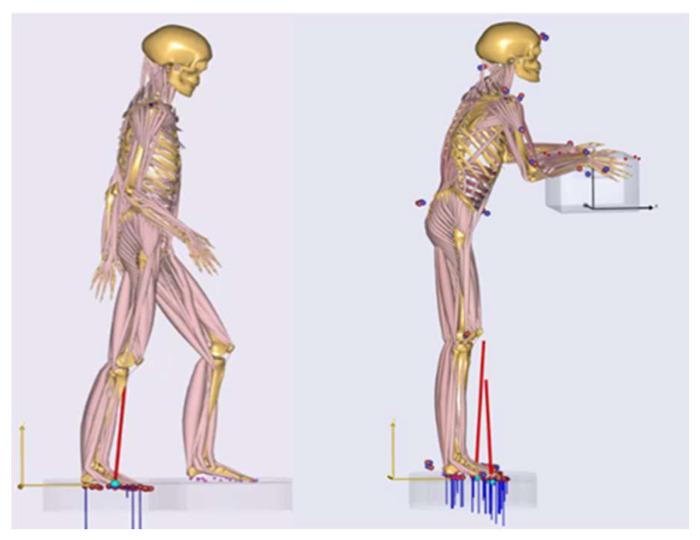
Modeled approaches for reference load scenarios as walking and holding a box. The joint force and moment magnitudes were taken within visualized postures in the walking model from the right knee and box holding model.

In discussion with ergonomics and biomechanical experts of the department Biomechatronic Systems of the Fraunhofer Institute representative muscle groups and joint loads were chosen and analyzed for each posture-representative model. To reference the computed joint loads natural biomechanical activities focusing on a specific body region where considered. Joint loads were determined by using simulation results and quantities of in vivo force measurements [20]. Figure 5 left shows the gait model in AMS, which quantified reference knee compressions for walking with a normal speed. The model for the calculation of the kinetic elbow and back references as lifting a 20 kg box in an upright standing posture is illustrated in Figure 5, left. The reference musculoskeletal models were both a 50th percentile European male. Ground reaction forces were predicted by the AMS [21] (see Figure 5). Further, the setup of the in vivo measurement of [20] where the participant performed a shoulder abduction of 45° with 2 kg weight in order to quantify the resultant force in the glenohumeral joint (GHJ) was taken as shoulder load reference. Muscle activities are referenced in their percentage distributions.

However, as relevant considered biomechanical output values are summarized and analyzed in an evaluation matrix. Based on the analysis excessively strained body regions can be leveled and outlined for the assistance potential of an exoskeleton.

### 2.3. Model-Based Exoskeleton Design Optimization and Evaluation

An exoskeleton system works cooperatively together with the human musculoskeletal apparatus. The interaction between the exoskeleton and the human body determines whether or how the exoskeleton can assist the desired movements. The human–exoskeleton system consists of two modules: (I) a musculoskeletal human body and (II) an exoskeleton model. The exoskeleton model contains all segments, joints, passive elastic elements and motors of the exoskeleton. The two modules are connected and form a single mechanical system in the analysis model.

Iteratively, the design of the exoskeleton can be improved by changing specific design parameters of the exoskeleton.

In a research project at Aalborg University, the model-based optimization approach, e.g., was applied for a passive shoulder orthosis to adapt spring stiffnesses for lowering muscle activities of the shoulder complex [22]. Further research studies, which follow the same approach, can be found [23,24,25].

**Figure 6:**
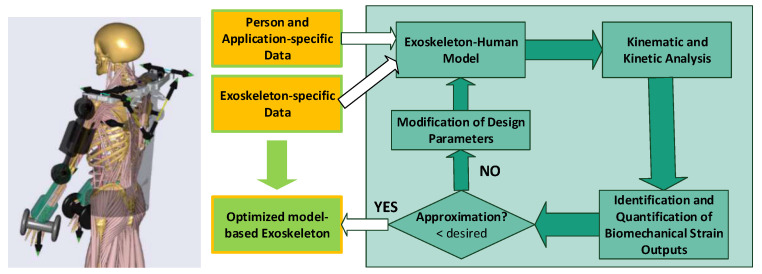
Exoskeleton–human model in AnyBody Modeling System (AMS) and model-based optimization loop. The optimization loop describes the different steps of implementing person and application-specific information as anthropometric, motion and force data. Further, an exoskeleton–human model in AMS is built to compute kinematic and inverse dynamic analysis to identify and quantify biomechanical output values. Compared with recommended ergonomic strain limit values, it is decided whether to abort or to continue with modifications of the any design parameters. The left exoskeleton–human model visualizes the Stuttgart Exo-Jacket, a potential supportive vest for manual carrying, holding and lifting tasks.

**Figure 7:**
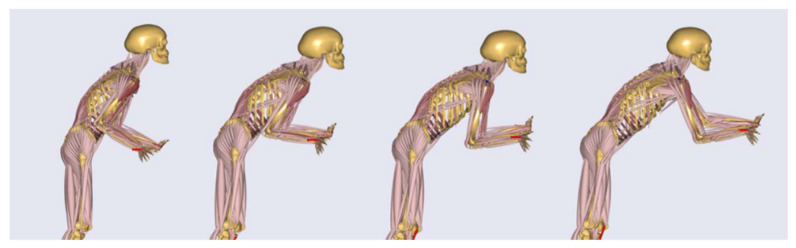
Dynamic human model in push movement illustrated as a sequence of images from the beginning to the end of the pushing. The motion is taken from a captured movement sequence, however it is subjectively defined as being representative of the movement.

Based on a preliminary exoskeleton concept, person and application specific data, a model-based optimization loop (see Figure 6) was applied. In this work, the push scenario with the highest computed load, as a short movement sequence, was taken as application specific data input for the optimization. The load scenario was selected based on aforementioned biomechanical analysis. Further the movement sequence was created by widening the static posture to a short movement of 1.5 s (see Figure 7) around the most loaded body posture. Motion and force data were extracted from lab measurements focusing on the highest force magnitudes of all test subjects in all trials to focus on biomechanical load peaks for the optimization.

Within the described dynamic modeling framework, the mechanical exoskeleton concepts were implemented and iteratively optimized.

**Figure 8:**
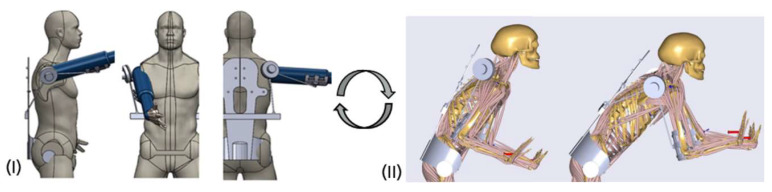
Virtual design frameworks for testing exoskeleton mechanisms (**I**): static human model and (**II**): dynamic multi-body simulation environment.

The concepts were optimized within two modeling frameworks: (I) a static exoskeleton–human designenvironment and (II) a musculoskeletal modeling framework, which are both considering conceptual exoskeleton designs. Within the digital frameworks virtual static range of motion tests (Figure 8 left) and dynamic tests with anatomic movements in a biomechanical more sophisticated framework (Figure 6 right) are executed.

The exoskeleton mechanism design is iteratively optimized by adapting design parameters in the CAD environment, certifying the range of motions and further proving biomechanical effects in the second framework and vice versa. After the exoskeleton CAD-design is optimized by certifying the range of motion of the mechanics and increasing the biomechanical effectiveness, the next step in the model-based workflow is the evaluation of the exoskeleton in order to investigate the overall biomechanical impact of the device on the musculoskeletal apparatus of the human body. It is important to guarantee that the designed exoskeleton mechanics supports the body in a proper way without leading to an increased loading of surrounded tissue and segments of the human body. Therefore, the muscle activations and joint forces are compared for the movement with and without the designed exoskeleton in AMS. In order to interpret the data in an ergonomic way, a baseline or reference value of the musculoskeletal loading are considered. Therefore, recommended hand forces of the German “Mounting-specific Force-Atlas” [26] applied on the 15th male percentile were used to generate reference values for an acceptable loading during the examined movement. A work planner normally uses this method to make sure that above the 15th male percentile every worker can execute a force-intensive work task. The evaluation method generates recommended maximum hand reaction forces, which should not be exceeded and which are taken as boundary conditions to compute references for acceptable body loadings. This approach is applied to the most relevant load scenario.

## 3. Results

**Figure 9:**
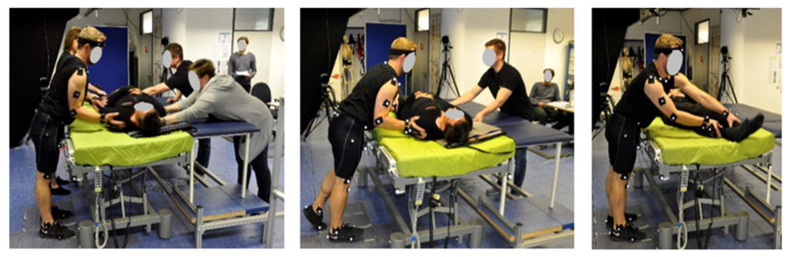
Manual handling tasks reproduced in the motion lab (left: pushing in a team; middle: pushing with less help and right: manually lifting the patient´s legs).

The workshop participants unanimously identified manual patient handling in the surgery waiting room as the most critical scenario from the ergonomic point of view. Three tasks were chosen for further analysis, namely (1) pulling and (2) pushing of a fully narcotized patient as well as (3) lifting the patient’s legs without his help from the edge of the bed and backwards (see Figure 9 from left to right).

### 3.1. Subjective Feedback of Perceived Body Strain

Depending on the situation and availability, either two or four caregivers handle the patient transfer in the waiting room, with either one or two persons involved on each side of the beds. Subjective feedback was therefore collected in both cases for the pushing and pulling tasks to investigate the influence of this organizational aspect on the perceived physical strain. On the other hand, lifting the patient´s legs from one side of the bed to another during op-preparation is normally performed by a single caregiver.

**Figure 10:**
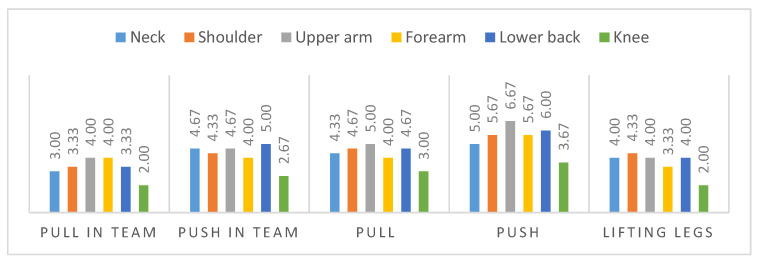
Results of subjective perception for application scenarios. “Pull in Team” and “Push in Team” refer to the cases where four caregivers are involved, while for “Pull” and “Push” they are only two.

The questionnaire collects subjective perceived strain in a score from zero to eight (less to strong strain) for each body region. Figure 10 illustrates the average results of the three subjects within two trials for each scenario. The results indicate high strains for the lower back (maximum score of 6.0) and the shoulder–arm-system, represented by the upper arm strain (maximum score of 6.67). In comparison to the lower extremities, represented by the knee strain, that were perceived as less loaded. The highest perceived strain occurred in the upper arm during pushing alone and in a team.

### 3.2. Model-Based Biomechanical Analysis

For the model-based analysis, each body region´s most representative muscle and joint output values were analyzed. Table 1 and Table 2 give an overview of the results, allowing the identification of the most loaded body load parameters. For the model-based analysis, only the scenarios executed alone by the subjects were considered to draw a baseline for most loaded postures.

The figures indicate high body strains for the shoulder area for all scenarios and high strains for the lower back mainly for the lifting posture. As the push scenario yielded critical values (the highest values for each posture, marked in red in the tables) for both muscle activity and joint compression forces in the shoulder area, it was chosen as basis for the optimization of the exoskeleton design.

### 3.3. Model-Based Exoskeleton Design Optimization

Based on the results of the previous analysis, different exoskeleton design concepts were generated and investigated with the goal to reduce musculoskeletal loading for the shoulder–arm system in the push scenario. One of these concepts is described in this article to demonstrate the model-based optimization.

Due to the mainly horizontal hand forces during patient transfer and the shoulder as the body region with highest loading, various mechanisms with one shoulder actuator were considered to support anteversion and retroversion of the shoulder during pushing and pulling activities. After a detailed analysis of the key functionalities and requirements for a shoulder–arm exoskeleton, one mechanism was selected from several conceptual drafts as the most promising using a selection matrix with weighted criteria as subjectively perceived by the developers, including overall weight, range of motion, collision probability with wearer, technical complexity, controllability, usability and biomechanical effectiveness.

**Figure 11:**
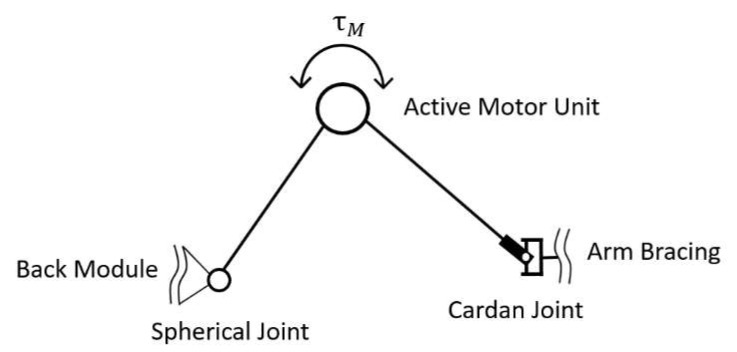
Mechanical exoskeleton concept to generate push and pull support.

The main characteristics of the chosen mechanism (see Figure 11) are a folding mechanism with an active revolute, a spherical and a universal passive joint mechanically connecting a back module with an upper arm bracing. Mainly presumed, the mechanism transfers forces along a line between both human interfaces.

**Figure 12:**
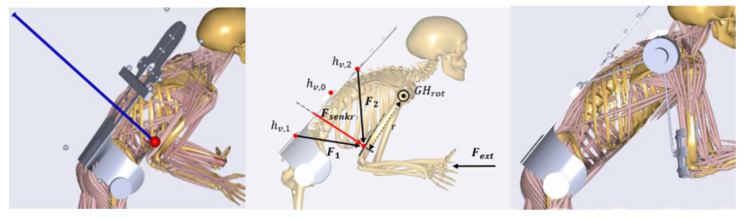
Exoskeleton–human models with outlined artificial force in blue for abstract optimal support estimation (**left**), varying back module attachment locations for mechanism to find optimal configuration (**middle**) and final concept with implemented design (**right**).

After the selection, the mechanism passed through several design steps from a conceptual draft to a conceptual CAD-design leading to an exoskeleton–human model, which is shown in Figure 12. The shoulder–arm mechanism was then modeled in the AMS within the aforementioned dynamic pushing movement sequence. As optimization parameters of the mechanism the attachment point of the shoulder–arm mechanism relative to the back module and the amount of torque support exerted by the active motor unit were considered. The reaction force in the GHJ was chosen as the biomechanical target variable for the optimization process.

Within two optimization iterations on different levels of modeling abstractions, the attachment point was located to facilitate an optimal mechanical support by the exoskeleton mechanism. The preliminary study exerted a conceptual artificial force between the upper arm bracing and the back module to find out in what geometrical direction the mechanism should be positioned for optimal biomechanical assistance. The analysis figured out that the mechanism has to be placed roughly at the height of the upper lumbar spine (see left image of Figure 12). In a further analysis, the attachment point was identified more specifically within a movable exertion point (see illustration in the middle of Figure 12).

**Figure 13:**
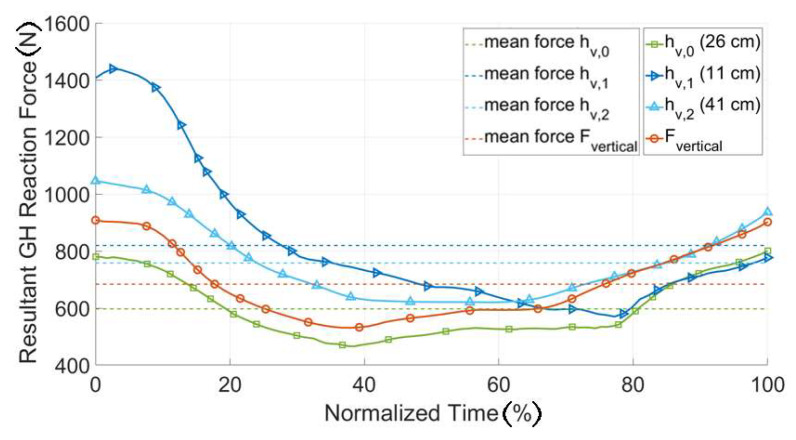
GHJ reaction forces for different attachment point configurations, the solid line represents the continuous joint force during the movement sequence. The dotted lines represent the mean force magnitudes for all attachment configurations.

Figure 13 shows that the position of the attachment point (h) greatly influenced the magnitude of the resultant GHJ reaction force, especially in the initial phase of the push motion (normalized time < 20%). In the later phases the sensibility to this parameter decreased and at normalized time = 60%, the reaction force was nearly the same for all the parameter values. The attachment configuration with the lowest average joint reaction force over the sequence was the configuration with an attachment point at 26 cm, measured from the bottom of the back plate and was selected as the biomechanically most effective position (Figure 12 right).

**Figure 14:**
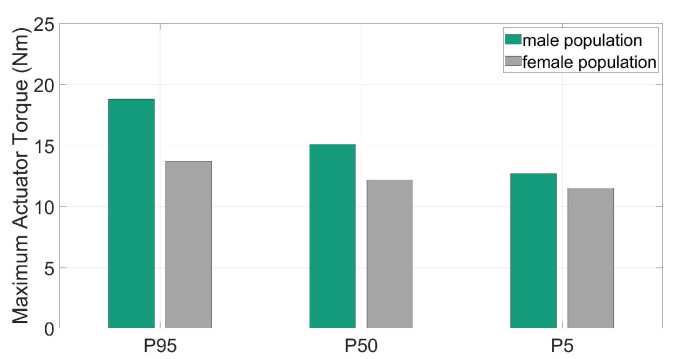
Maximal support torque values for representative individuals of male and female.

To define the requirements for the exoskeleton drive module a further optimization was applied to find the amount of supportive torque needed for the design load scenario. The torques were quantified with AMS for different populations with variable recommended maximum hand forces for the specific movement based on from occupational ergonomics experts recommended forces [26]. These are represented in Figure 14 for male and female individuals (5th, 50th and 95th percentiles) together with the corresponding hand reaction forces.

The supportive motor torques varied between 12.7 and 18.8 Nm for men and 11.5 and 13.7 Nm for women (see Figure 14). These results formed the basis for the design of the active motor unit. It should be considered that the gravity of the motor unit was more relevant in less actively supported scenarios.

Iteratively within the kinetic optimization of the designed shoulder–arm exoskeleton, its range of motion was analyzed to identify possible kinematic restrictions. Achievable shoulder anteversion, retroversion, abduction and adduction angles were evaluated using a human CAD model. The result of the kinematic analysis outline a maximum anteversion angle of 105° and a maximum retroversion angle of 10°, which means that the mechanism will rather marginally restrict the wearer´s range of motion in the entire 120° range of motion coming from the lab analysis. Furthermore, the backwards movement of the humerus was slightly restricted, due to the attachment point at the back module. Maximum angles for adduction and abduction were at 7° and 90°, which means no restriction for all three analyzed movement sequences.

### 3.4. Model-Based Exoskeleton Design Evaluation

**Figure 15:**
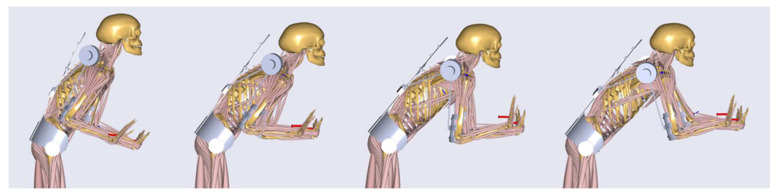
Musculoskeletal evaluation exoskeleton–human model in the push movement sequence.

After the optimization of the designed shoulder–arm mechanism, the exoskeleton was evaluated in order to investigate its biomechanical effectiveness and impact on the whole human body. For that purpose, the case of the pushing task with the highest external hand forces over all three participants (250 N) and an actuator torque of up to 30 Nm were used. Single frames of the simulated dynamic motion with the starting, two middle and the ending pose are shown in Figure 15. Here the resultant GHJ forces and the muscle activity of the deltoid were taken as the most representative biomechanical outputs to represent the loading of the shoulder–arm system.

**Figure 16:**
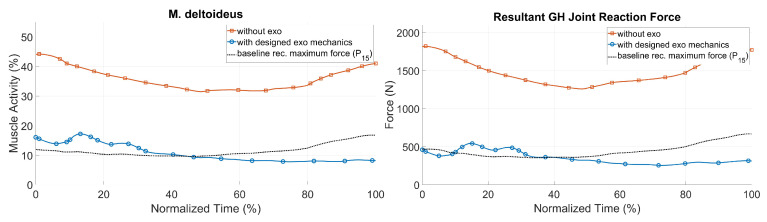
Biomechanical results for the push scenario for the muscle and joint loads of the right shoulder.

**Figure 17:**
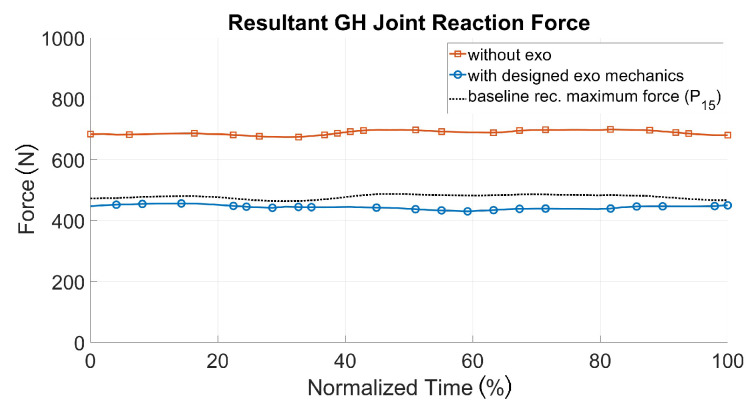
Representative joint loads of the right shoulder for the pull scenario with (blue line) and without the exoskeleton (red line) in comparison with recommended manual pulling (dotted black line) [26].

Figure 16 shows the simulation results for the two biomechanical output values during the pushing task with and without the exoskeleton, together with the baseline of recommended hand forces for the 15th percentile [26] for an acceptable loading. The GHJ force was significantly reduced with the optimized shoulder–arm exoskeleton: its maximum value was decreased from 1821 to 573 N, which corresponded to a drop of more than 70%. The average muscle activity of the deltoid was also reduced by more than 25% during the same sequence. As a result, the simulated exoskeleton support led to a significant decrease in shoulder loading, up to a range of values comparable to those of the baseline for acceptable loadings. The strain of the shoulder complex in the pull scenario could be decreased as well (see Figure 17). Therefore, a recommended pull force of 52.5 N [26] for each hand was taken as an external force for modeling reference purposes, in comparison to 70 N (each hand) for the patient handling scenario with and without the exoskeleton.

Besides shoulder loading, other key biomechanical outputs were investigated to get a holistic view of the impact of the exoskeleton use, which are illustrated in Table 3. These include the global metabolic power of all active muscles, the activation of stabilization muscles of the rotator cuff and parameters as joint or muscle loading of additionally affected body regions, like the neck, chest and back. It shows that the metabolic power of all muscles is decreased by more than half when integrating the designed exoskeleton. It also reduces largely the forces in the acromioclavicular and sternoclavicular joints, as well as the activation of the muscle linked to motion generation and joint stabilization. On the other hand, a slight increase of the activities of the trapezius and pectoralis muscles is observed compared to the case without exoskeleton. Although the difference was only 2%, this issue is a necessary topic of future investigations in order to avoid an increase of loadings and to understand such effects. Further, the huge decrease of the lower back outputs with an exoskeleton should be further understood, the exoskeleton model’s back module has shoulder straps, which are connected to a stiff back plate. More realistic modeling of the back plate will be surely a worthwhile focus for future research.

In order to evaluate the exoskeleton–human interaction, pressure forces at the human–exoskeleton interfaces were further analyzed. The resultant pressures on these contact regions were calculated by the sum of the simulated interaction forces between the exoskeleton and human in estimated contact areas. The computed average interface pressures for the pelvis, thorax and upper arm regions were all under maximum comfort level (maximum pressure of 9.3 kPa) based on the maximal recommended values for pressure ulcer prevention [27].

## 4. Discussion

Regarding the development of systems that physically support the human body most studies are based on a conceptual design [8]. In comparison to those studies, the methodology of this work focused on the application-centered exoskeleton designing using a real environment analysis and real workers motion data in a realistic mock-up. With this approach, it is possible to design a system more specifically and to determine biomechanical benefits practically for selected loading scenarios. One additional key aspect of this work is that biomechanical loadings of the musculoskeletal system during the pulling and pushing scenarios are compared to reference loadings, which are based on recommended manual work force data.

The exoskeleton design illustrated in this paper shows high potential for assisting in manual pushing and pulling. Nevertheless, there is still much work to optimize its effectiveness considering more applications. Further, it is necessary to investigate the positioning of the attachment point of the spherical joint at the back module for different percentiles. Based on the results, height-dependent positioning can be determined to increase the biomechanical effectiveness of the designed shoulder–arm mechanism. With those studies, it will be possible to individualize mechanical components for specific user groups and individual percentiles.

The described mechanics is evaluated for one subject model within two movement sequences, this should be made clear to interpret the studies’ evaluation results. Further investigations with more person-specific models would further increase evaluation entirety. The modeling complexity limited the number of investigated coupled exoskeleton–human models.

Further, the motion data could be captured in future studies using field applicable inertial motion sensor suits. Laboratory mock-ups are recommended for person-specific research purposes of wearable assistive devices to ensure accurate and reproducible movement and load boundary conditions.

The combined kinetic exoskeleton–human modeling approach is highly recommended for investigating the biomechanical effects of wearable assistive devices. The approach quantifies objective body load quantities in comparison to more subjective biosignal measurements of, e.g., muscle activities (surface electromyography) or oxygen consumption (ergospirometry), which are from ergonomists traditionally recommended. Especially for holistic ergonomic evaluation purposes the digital model-based analysis approach seems to be promising for improved understanding of the effects of *occupational exoskeletons* during the hole working procedure [28].

The mechanical exoskeleton–human interfaces have been restrictedly considered in this study to ensure acceptable interaction forces and contact pressures. The modeling approach would further enable more sophisticated mechanical interface analysis to potentially increase haptic comfort for the wearer.

## 5. Conclusions 

For future work, it is necessary to investigate mechanical configurations that lead to higher loadings of single muscle groups (e.g., M. trapezius with 2% higher loading with the implementation of the exoskeleton). Analyses that are more detailed are necessary in order to prevent an undesired increase of loading on the one side and to get an improved understanding of the impact of mechanics on the human body on the other side.

**Figure 18:**
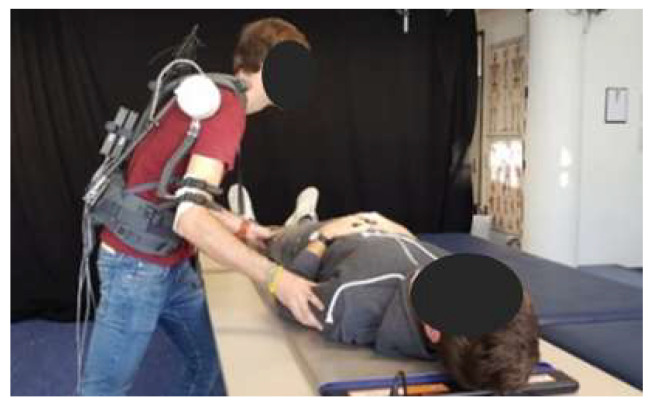
Preliminary laboratory tests with a prototype of the investigated exoskeleton design during the patient push scenario.

The digital optimization and evaluation approach illustrated in this paper shows high potential for developing, adjusting and evaluating individualized assistive devices. However, there is still much work to optimize its effectiveness. Possible improvement for future works pursuing the presented model-based methodology is the use of dynamic multi-body models for kinematic design. The utilized static CAD human model connected with the CAD mechanics only allows for the testing of single (maximum) postures. Dynamic multi-body models are recommended in order to design mechanisms in a more dynamic way and to identify possible kinematic restrictions and challenges much earlier in the developing process. Especially for mechanisms that are more complex it is suitable to separate kinematic analysis regarding the range of motion and the kinetic modeling using sophisticated biomechanical human models. A prototype for the exoskeleton concept is currently under development (see Figure 18) and was preliminary tested in the laboratory application mock-up by team-internal developers. The feedback was quite well, but will be extended by tests with real surgery assistants.

Since the modeling of the human–exoskeleton interface using digital human models is a relatively new field of research, it is necessary to validate the simulated results in further validation studies. For that purpose, a prototype of the exoskeleton design presented in this paper is as aforementioned currently under development. Using this exoskeleton as an investigative tool, the validity and accuracy of the proposed digital analysis approach can be investigated using different methods. One possibility is to compare real muscle activities with simulated ones during exoskeleton wearing by using surface electromyography. Another method would consist of measuring the interaction forces between segments of the human body and exoskeleton mechanics with force sensors. Model accuracy could then be determined by comparing the quantitative trend of the measurements with the model predictions.

Beside the illustration of applicability of the proposed design of a wearable assistive device, the results presented here also show the potential of a simple active exoskeleton to reduce the caregivers’ biomechanical loads. This will lead to a more ergonomic manual handling of heavy narcotized patients in the surgery waiting room and potentially decrease the incidence of WMSDs in the healthcare sector.

## Figures and Tables

**Table 1:** Maximal muscle activities overall body regions.

Body Region	Neck	Upper Arm			Forearm			Lower Back	Lower Extremities	
Muscle Groups	M. Trapezius	M. Deltoideus (left) M. triceps Brachii (middle) M. Biceps Brachii (right)			M. Extensor Carpi (left) M. Flexor Digitorum (right)			M. Erector Spinae (Sacrum)	M. Biceps Femoris (left) M. Semi-Tendinosus (right)	
Modeled Quantity	Activity (%)	Activity (%)			Activity (%)			Activity (%)	Activity (%)	
Push Posture	4	56	7	**58**		26	0	28	23	19
Pull Posture	19	19	18	5		5	3	**39**	36	33
Lifting Legs Posture	54	34	0	25		16	20	**55**	20	20

**Table 2:** Maximal joint reactions overall body regions.

Body Region	Ellbow		Shoulder		Lower Back		Knee	
**Modeled Quantity**	Flexion Moment		Forces in GHJ		Compression Forces in L4/L5		Flexion Moment (left) Compression Forces (right)	
Push Posture	3.1 Nm	11%	**1405 N**	**163%**	2087 N	116%	27 Nm	1225 N
Pull Posture	−3.5 Nm	12%	**1305 N**	**151%**	1162 N	65%	69 Nm	3555 N
Lifting Legs Posture	21 Nm	72%	1250 N	145%	**2775 N**	**154%**	72 Nm	4067 N
References	29 Nm	100%	863 N	100%	1800 N	100%	75 Nm	4087 N

**Table 3:** Overview of the influence of exoskeleton use on key biomechanical parameters for the pushing task in comparison to those with an exoskeleton, without an exoskeleton and without an exoskeleton and recommended pushing force quantity.

Area	Unit	Without Exo	With Exoskeleton	Baseline P15
**Metabolic Parameter**				
Total Metabolic Power	W	1120	460	512
**Shoulder–arm Apparatus max. Values**				
Envelope ACJ SCJ force ^1^	N	1092	654	309
M. deltoideus	%	44	18	17
M. biceps brachii	%	50	12	15
Envelope rotator cuff	%	44	13	17
**Neck and Chest Muscles max. Values**				
M. trapezius	%	16	18	9
M. pectoralis	%	12	2	3
**Lower Back max. Values**				
M. erector spinae	%	52	12	28
L4/L5 compression force	N	2358	1478	1750

^1^ Envelope of joint reaction force of acromioclavicular joint (ACJ) and sternoclavicular joint (SCJ).
